# Association between the dietary obesity-prevention score and risk of gestational diabetes mellitus: an exploratory subgroup analysis

**DOI:** 10.3389/fnut.2026.1869787

**Published:** 2026-07-24

**Authors:** Ying Xie, Hongmei Xue, Sijie Zhou, Liting Li, Dandan Wang, Yan Guo, Jianqiang Lai, Zengning Li

**Affiliations:** 1 School of Public Health, Hebei Medical University, Shijiazhuang, China; 2 Department of Clinical Nutrition, The First Hospital of Hebei Medical University, Shijiazhuang, China; 3 Central of Laboratory, The First Hospital of Hebei Medical University, Shijiazhuang, China; 4 Hebei Key Laboratory of Nutrition and Health, Shijiazhuang, China; 5 Department of Radiology, The First Hospital of Hebei Medical University, Shijiazhuang, China; 6 National Center for Rural Water Supply Technical Guidance, Chinese Center for Disease Control and Prevention, Beijing, China

**Keywords:** cohort study, dietary obesity-prevention score, dietary pattern, fruit intake, gestational diabetes mellitus

## Abstract

**Background:**

Gestational diabetes mellitus (GDM) presents significant risks to maternal and offspring health. Modifiable factors like dietary quality are important for prevention. The Dietary Obesity-Prevention Score (DOS) is a validated tool for assessing obesity-related dietary quality. Nevertheless, its association with GDM remain unclear. This study aimed to examine the relationship between DOS and GDM risk.

**Method:**

This prospective cohort included 1,894 pregnant women across nine medical centers in China. Dietary intake was assessed by 3-day 24-h recalls. The DOS was derived from 6 food groups. Multivariable binary logistic regression, component analysis, subgroup analysis, and restricted cubic splines (RCS) were used to evaluate associations.

**Results:**

In the overall population, DOS showed no significant association with GDM (OR = 0.944, 95% CI: 0.879–1.015). The interaction between DOS and energy intake status was not significant (*p* for interaction = 0.193). Exploratory subgroup analyses showed that among women with inadequate energy intake, higher DOS was associated with reduced GDM risk (OR = 0.867, 95% CI: 0.773–0.973). Likelihood ratio tests showed that excluding fruit from the full model worsened the model fit (Δ-2LL = 16.091, df = 1, *p* < 0.001). Component analysis suggested a possible inverse association between fruit intake and GDM risk (OR per 50 g/1,000 kcal = 0.905, 95% CI: 0.817–1.001, *p* = 0.053). Fruit intake was associated with lower GDM risk in the middle DOS tertile (OR = 0.783, 95% CI: 0.620–0.990, *p* = 0.041) and showed a borderline significant association in the high DOS tertile (OR = 0.828, 95% CI: 0.686–1.000, *p* = 0.049). In the inadequate energy intake subgroup, RCS analysis revealed a non-linear association between fruit intake and GDM risk (*p* for non-linearity = 0.048), with risk decreasing to a nadir at approximately 400 g/day, after which it stabilized. The 95% CI widened beyond 600 g/day due to sparse data, and these inflection points should be interpreted as exploratory descriptive features rather than precise thresholds.

**Conclusion:**

In pregnant women with inadequate energy intake, higher dietary quality was associated with reduced GDM risk. Fruits may play a role, although these findings are exploratory and require confirmation in independent cohorts.

## 1 Introduction

Pregnancy is a distinct physiological period during which both overnutrition and undernutrition can adversely affect maternal and fetal health. Gestational diabetes mellitus (GDM) is defined as glucose intolerance with onset or first recognition during pregnancy. It is one of the most common metabolic complications of gestation. GDM increases the risk of adverse perinatal outcomes. These include maternal metabolic disturbances, preeclampsia, preterm birth, and cesarean section (1–3). For offspring, GDM is associated with macrosomia, neonatal hypoglycemia, and long-term metabolic disorders (4, 5). In recent years, with the improvement of living standards, dietary habits and trends have changed. People tend to choose ultra-processed foods with high energy and low nutrient density, and the high sugar, high salt, and high-fat diet patterns are becoming increasingly prominent (6). The incidence of diabetes during pregnancy is increasing year by year. The global burden of GDM is substantial. According to the International Diabetes Federation, the standardized prevalence of GDM is estimated at 14.0% based on the International Association of the Diabetes and Pregnancy Study Groups (IADPSG) criteria (7). The prevalence of GDM in China is also a cause for concern. A meta-analysis indicated that the prevalence of GDM after 2010 has reached 15.6%; moreover, according to another study, the prevalence has increased sharply from 4% in 2010 to 21% in 2020, demonstrating a rapidly growing trend (8, 9). Given its rising prevalence and dual impact on maternal and child health, effective strategies for GDM prevention are needed.

Dietary modification plays an important role in GDM prevention and management (10). However, current dietary assessment tools have certain limitations. Commonly used diet quality indices are narrow in scope and built on outdated food components, particularly neglecting ultra-processed foods (UPFs), which have been increasingly recognized as a risk factor for adverse metabolic outcomes (11). The dietary Obesity-Prevention Score (DOS) is a food-based assessment tool that evaluates the relationship between individual dietary quality and obesity prevention by rating 14 groups of foods (with an overall DOS score potential range of 14–42) (12). In non-pregnant populations, higher DOS has been associated with lower risks of overweight and obesity, less annual weight gain, and improved cardiometabolic profiles. These include lower blood pressure and favorable lipid levels (13, 14). Higher DOS has also been linked to a reduced risk of type 2 diabetes (15). However, no significant association was observed between DOS and polycystic ovary syndrome (16).

Collectively, these findings suggest that DOS is a promising dietary pattern for metabolic health. The established benefits of DOS in the general population are supported by current research. However, unique metabolic changes are present during pregnancy, and these changes differ from those in the non-pregnant state. But given that excessive gestational weight gain is a major driver of GDM, the architecture of DOS is considered suitable for the identification of obesogenic dietary patterns, which are regarded as crucial during pregnancy. Therefore, this study was designed to explore the association between DOS and GDM. Furthermore, the potential pathways through which a high-quality diet might influence GDM are unknown. These may include increased intake of specific food groups, such as fruits, vegetables, or plant protein. Therefore, this study was conducted to examine the association between the DOS and GDM risk in pregnant women, and to assess whether this association is modified by energy intake status. This study further aims to identify key foods that play an important role and explore their dose-response relationship.

## 2 Materials and Methods

### 2.1 Study design

**Figure 1:**
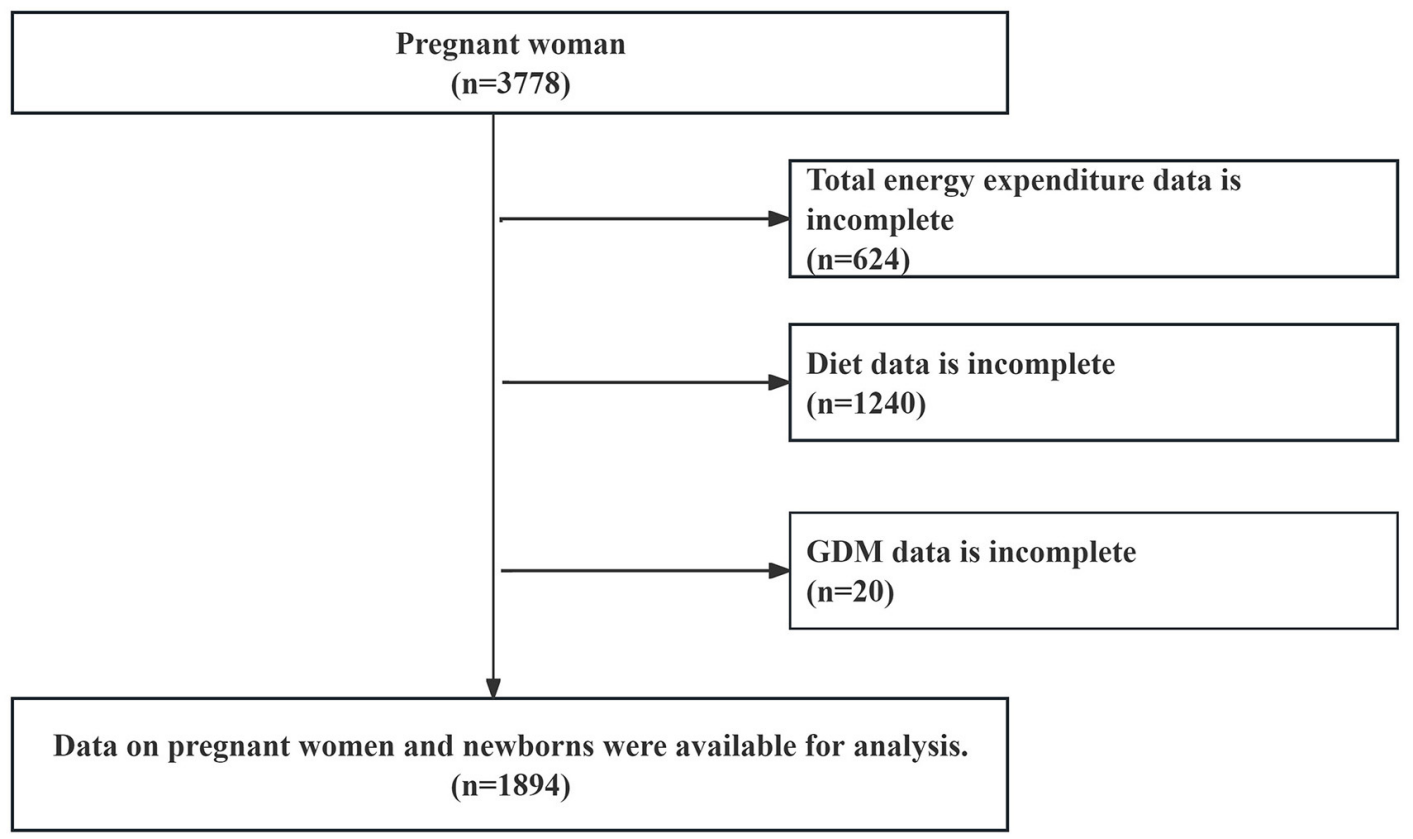
Flow chart of participant selection.

The data for this study were derived from a mother-infant birth cohort collected by a research team led by the Chinese Center for Disease Control and Prevention between March 2013 and May 2014. Participants were sourced from nine centers. Pregnant women at 6–12 weeks of gestation were enrolled in this cohort. Participants with a history of kidney stones, renal insufficiency, abnormal thyroid function, gastrointestinal diseases, lung disease, HIV infection, active tuberculosis, diabetes mellitus, dementia, anemia, mental disorders, or those who reported experiencing threatened abortion were excluded from the study. After enrollment, pregnant women completed three rounds of questionnaire surveys to provide information on demographic characteristics, diet, behavior, and other factors. Physical measurements were also conducted at each visit. A total of 3,778 participants were included in this study. After excluding participants with missing data necessary for calculating total energy expenditure (TEE), incomplete dietary data, implausible total energy intake (<500 or >6,000 kcal/day), and incomplete GDM data, a total of 1,894 participants were included (Figure 1) (17, 18).

### 2.2 Dietary assessment and calculation of DOS

Dietary intake was assessed using a 3-day 24-h dietary recall method (including 2 weekdays and 1 weekend day) administered by trained interviewers during each trimester of pregnancy. Given the potential instability of dietary intake during the first trimester due to pregnancy-related factors such as nausea and vomiting, dietary data from the second trimester were used for the main analyses. Based on the Chinese Food Composition Table, average daily intakes of total energy (kcal/d), protein (g/d), fat (g/d), carbohydrate (g/d), and dietary fiber (g/d) were calculated for each trimester (19).

The DOS includes 14 food groups, but the alcohol consumption during pregnancy was negligible in this population. So this study calculated the score based on 13 food groups. Foods were classified into seven positively weighted and six negatively weighted categories. First, using the residual method, daily intake (g/d) of each food group was energy-adjusted, and tertiles were assigned. The DOS was calculated by summing the tertile scores for positively weighted food groups and the reversed tertile scores for negatively weighted food groups. Second, the energy-density method was applied, where intake per 1,000 kcal was calculated for each food group and tertile scores were assigned analogously. Given that dietary recall has limitations and cannot accurately capture episodically consumed foods. Missing food items do not always represent true zero intake, and zero imputation may introduce bias (20). Based on distributional considerations and with priority given to food groups that are easily recalled for clinical use, rarely consumed food groups with >50% missing values, and then those with >30%, were excluded. Multiple DOS versions were constructed following predefined rules. Detailed compositions of each version are provided in Supplementary Table S1. The final DOS was constructed using the energy-density method and comprising vegetables, fruits, plant to animal protein ratio, and reverse-scored red meat, refined grains, and ultra-processed foods. DOS showed a low correlation with total energy intake (Supplementary Figure S1). The association between DOS and GDM risk was compared with that of the DASH score, an established dietary index, to contextualize the findings. The DASH score was calculated using two versions: DASH-7 (seven components) and DASH-8 (eight components). Detailed methods are provided in Supplementary Table S2.

### 2.3 Anthropometric assays

Height and pre-pregnancy weight were obtained from baseline interviews conducted at 0–13 weeks of gestation. Height was measured in meters (to two decimal places), and weight in kilograms (to one decimal place), as recalled by the participants and verified by trained staff. Body weight was measured at 12 and 28 weeks of gestation. Body mass index (BMI) was calculated as weight (kg) divided by height squared (m^2^). Pre-pregnancy BMI was categorized according to Chinese criteria: underweight (<18.5 kg/m^2^), normal weight (18.5 ≤ BMI <24.0 kg/m^2^), overweight (24.0 ≤ BMI <28.0 kg/m^2^), and obese (≥28.0 kg/m^2^) (21).

### 2.4 Diagnosis of GDM

GDM was diagnosed according to IADPSG criteria. A 75-g oral glucose tolerance test (OGTT) was performed between 24 and 28 weeks of gestation. GDM was defined as having one or more of the following plasma glucose thresholds met or exceeded: fasting glucose ≥5.1 mmol/L, 1-h glucose ≥10.0 mmol/L, or 2-h glucose ≥8.5 mmol/L (10).

### 2.5 Total energy expenditure

Total energy expenditure (TEE) was estimated as basal metabolic rate (BMR) multiplied by physical activity level (PAL). PAL refers to the method recommended by Fayet-Moore et al. (22). This estimate was used as the individualized energy requirement to avoid uncertainties associated with gestational weight gain recommendations. The energy intake ratio was calculated as energy intake during the second trimester divided by TEE. Insufficient intake was defined as <90% of the requirement, sufficient as 90%−100%, and excessive as >110% (23).

BMR was calculated using the Mifflin-St Jeor equation:


$$\begin{array}{l} BMR\;\left(kcal/d\right)=10\times weight\;\left(kg\right)+6.25\times height\;\left(cm\right)\\ \;-5\times age\;\left(years\right)-161\\ \;TEE=BMR\times PAL\; \end{array}$$

### 2.6 Covariates assessment

After enrollment, a structured questionnaire was administered to collect sociodemographic characteristics, anthropometric parameters, lifestyle factors, and biochemical test results. Trained researchers completed the questionnaire. Information was collected on maternal age, education level, occupation, per capita monthly income, family history, gravidity, parity, exercise habits before and during pregnancy, insomnia during the second trimester, and the husband's education level and occupation.

Education level was dichotomized as ≤ 12 years (senior high school or below) and >12 years (college degree or above). Occupation was classified as mental work or manual work. Per capita monthly income was categorized as <5,000 CNY or ≥5,000 CNY. Exercise habits before pregnancy and during the second trimester were recorded as “yes” or “no”. Insomnia during the second trimester was recorded as “yes” or “no”. Gravidity was grouped as ≤ 1 or >1. Parity was grouped as <1 or ≥1. Family history in first-degree relatives was categorized as “yes”, “no”, or “unclear”. Mode of delivery was classified as natural birth or cesarean section. The husband's education and occupation were categorized using the same definitions as for pregnant women.

Data were complete for most covariates, with a small number of missing values: per capita monthly income (*n* = 3 missing), exercise before pregnancy (*n* = 1), exercise during second trimester (*n* = 3), family history of diabetes (*n* = 3), thyroid disease (*n* = 5), hypertension (*n* = 1), hyperlipidemia (*n* = 2), simple obesity (*n* = 2), gravidity (*n* = 3), parity (*n* = 9), insomnia during second trimester (*n* = 5), and mode of delivery (*n* = 1). These missing data were handled by complete-case analysis in multivariable models, as the overall missing proportion was low (<1%).

### 2.7 Statistical analysis

Statistical analyses were performed using SPSS (version 26) and R software (4.5.2). Normality of continuous variables was assessed using the Shapiro-Wilk test. As variables such as age were not normally distributed in certain subgroups, all descriptive statistics were presented as medians with the 25th and 75th percentiles. Group comparisons for continuous variables were conducted using the Kruskal-Wallis test. Categorical variables were expressed as frequencies and percentages, and compared using the chi-square test. Multivariable binary logistic regression models were used to examine the respective association of the DOS and the DASH score (during the second trimester) with the risk of GDM. Models were adjusted for pre-pregnancy BMI, maternal age, and other covariates described in Section 2.6, including education, occupation, per capita monthly income, family history of diabetes, exercise before pregnancy, and the husband's occupation. Variables with missing data were handled by complete-case analysis. Analyze the contribution of a single food group to the model fitting through likelihood ratio test. To identify the primary contributor among food groups, component analysis was performed using a mutually adjusted model. Exploratory subgroup analysis was then conducted to investigate how the relationship between dietary quality and fruit intake influences GDM risk. Dose-response relationships were explored using restricted cubic splines (with 3 knots) in R, with the median intake used as the reference point. Sensitivity analysis using E-values was conducted for the association between fruit intake and GDM to assess robustness to unmeasured confounding (24, 25). All statistical tests were two-sided, and statistical significance was set at α = 0.05. Forest plots and restricted cubic splines were generated in R.

## 3 Results

### 3.1 Participant characteristics

A total of 1,894 pregnant women were included in this study. The median age of participants was 28.6 (IQR: 26.5–30.6) years. Baseline characteristics of participants stratified by energy intake status during the second trimester are presented in Table 1. Significant differences were observed across energy intake groups for husband's occupation, per capita monthly income, family history of diabetes and hypertension, pre-pregnancy BMI, rate of weight gain from 12 to 28 weeks, exercise habits before and during pregnancy, insomnia during the second trimester, and mode of delivery (all *p* < 0.05).

**Table 1:** Baseline characteristics of participants by energy intake status in the second trimester (*n* = 1,894).

Variable	Total (*n* = 1,894)	Inadequate (*n* = 729)	Adequate (*n* = 540)	Excessive (*n* = 625)	*p*-value
Demographics					
Age (years)	28.6 (26.5, 30.6)	28.6 (26.3, 30.6)	28.5 (26.5, 30.6)	28.6 (26.7, 30.7)	0.406
Education of pregnant woman (%)					0.404
≤ 12 years	282 (14.9)	114 (15.6)	71 (13.1)	97 (15.5)	
>12 years	1,612 (85.1)	615 (84.4)	469 (86.9)	528 (84.5)	
Education of husband/partner (%)					0.845
≤ 12 years	326 (17.2)	130 (17.8)	90 (16.7)	106 (17.0)	
>12 years	1,568 (82.8)	599 (82.2)	450 (83.3)	519 (83.0)	
Occupation of pregnant woman (%)					0.195
Mental work	1,056 (55.8)	405 (55.6)	317 (58.7)	334 (53.4)	
Manual work	838 (44.2)	324 (44.4)	223 (41.3)	291 (46.6)	
Occupation of husband/partner (%)					0.015
Mental work	1,229 (64.9)	502 (68.9)	340 (63.0)	387 (61.9)	
Manual work	665 (35.1)	227 (31.1)	200 (37.0)	238 (38.1)	
Per capita monthly income (%)^*^					<0.001
<5,000 CNY	856 (45.3)	271 (37.2)	242 (44.9)	343 (55.1)	
≥5,000 CNY	1,035 (54.7)	458 (62.8)	297 (55.1)	280 (44.9)	
Family history (yes, %)					
Diabetes^*^	262 (13.8)	122 (16.7)	70 (13.0)	70 (11.2)	0.004
Thyroid disease^*^	76 (4.0)	28 (3.9)	18 (3.3)	30 (4.8)	0.444
Hypertension^*^	500 (26.4)	215 (29.5)	144 (26.7)	141 (22.6)	0.033
Hyperlipidemia^*^	115 (6.1)	47 (6.4)	39 (7.2)	29 (4.6)	0.211
Simple obesity^*^	33 (1.7)	11 (1.5)	12 (2.2)	10 (1.6)	0.224
Gravidity (%)^*^					0.138
≤ 1	1,298 (68.6)	497 (68.3)	355 (66.0)	446 (71.4)	
>1	593 (31.4)	231 (31.7)	183 (34.0)	179 (28.6)	
Parity (%)^*^					0.822
<1	1,793 (95.1)	687 (94.8)	511 (95.5)	595 (95.2)	
≥1	92 (4.9)	38 (5.2)	24 (4.5)	30 (4.8)	
Pre-pregnancy BMI (kg/m^2^)	20.7 (19.1, 22.9)	21.3 (19.5, 23.8)	20.8 (19.2, 22.8)	20.0 (18.4, 21.8)	<0.001
Rate of weight gain from 12 to 28 weeks (kg/week)	0.5 (0.5, 0.7)	0.5 (0.4, 0.6)	0.5 (0.4, 0.7)	0.6 (0.4, 0.7)	<0.001
Regular exercise before pregnancy (%)^*^					0.003
Yes	273 (14.4)	86 (11.8)	73 (13.5)	114 (18.3)	
No	1,620 (85.6)	643 (88.2)	467 (86.5)	510 (81.7)	
Regular exercise during second trimester (%)^*^					<0.001
Yes	682 (36.1)	324 (44.6)	189 (35.1)	169 (27.0)	
No	1,209 (63.9)	403 (55.4)	350 (64.9)	456 (73.0)	
Insomnia during second trimester (%)^*^					<0.001
Yes	668 (35.4)	187 (25.8)	183 (33.9)	298 (47.8)	
No	1,221 (64.6)	538 (74.2)	357 (66.1)	326 (52.2)	
Pregnancy outcome					
Gestational diabetes mellitus (%)	275 (14.5)	113 (15.5)	76 (14.1)	86 (13.8)	0.624
Delivery gestational week (week)	39(38,40)	39(38,40)	39(38,40)	39(38,40)	0.088
Mode of delivery (%)^*^					<0.001
Natural birth	1,005 (53.1)	432 (59.3)	305 (56.5)	268 (42.9)	
Cesarean section	888 (46.9)	296 (40.7)	235 (43.5)	357 (57.1)	

Values are presented as median (25th−75th percentiles) for continuous variables and as *n* (%) for categorical variables. Group comparisons were performed using Kruskal–Wallis test for continuous variables and chi-square test for categorical variables. ^*^Indicates missing data.

DOS score and energy-adjusted dietary intakes according to energy intake status are shown in Table 2. The median DOS score was 12 (IQR: 11–13). With the exception of legumes and sugary beverages, all nutrient and food group intakes differed significantly across the three groups (all *p* < 0.05).

**Table 2:** DOS score and energy-adjusted dietary intake by energy intake status in the second trimester (*n* = 1,894).

Variable	Total(*n* = 1,894)	Inadequate (*n* = 729)	Adequate (*n* = 540)	Excessive (*n* = 625)	*p*-value
DOS	12 (11,13)	12 (11,14)	12 (11,13)	11 (10,13)	<0.001
Energy (kcal/d)	1,747.44 (1,454.20, 2,096.79)	1,374.70 (1,218.67, 1,499.49)	1,772.54 (1,666.99, 1,909.03)	2,198.14 (2,055.57, 2,325.35)	<0.001
Carbohydrate (g/d)	246.02 (199.45, 313.23)	193.47 (165.50, 218.20)	252.42 (222.06, 287.85)	328.74 (296.99, 357.04)	<0.001
Fat (g/d)	63.53 (50.25, 78.07)	49.27 (38.86, 58.85)	66.18 (56.54, 77.36)	79.01 (67.41, 92.43)	<0.001
Protein (g/d)	65.63 (55.46, 74.47)	53.75 (46.81, 61.51)	67.07 (60.83, 74.11)	74.46 (67.50, 84.24)	<0.001
Plant to animal protein ratio	1.17 (0.85, 1.58)	1.03 (0.76, 1.43)	1.10 (0.81, 1.53)	1.38 (1.05, 1.80)	<0.001
Vegetable to animal protein ratio	0.15 (0.10, 0.20)	0.15 (0.10, 0.22)	0.14 (0.10, 0.19)	0.15 (0.11, 0.20)	0.037
Fiber (g/d)	11.45 (9.14, 14.22)	9.55 (7.77, 11.83)	11.77 (9.46, 14.30)	13.42 (11.19, 16.05)	<0.001
Fruits (g/1,000 kcal)	157.70 (111.19, 225.86)	189.88 (127.84, 259.72)	157.55 (117.98, 224.15)	133.44 (98.56, 181.28)	<0.001
Vegetables (g/1,000 kcal)	184.12 (138.41, 242.59)	215.88 (159.52, 284.63)	183.95 (139.03, 235.75)	156.28 (123.24, 199.46)	<0.001
Legumes (g/1,000 kcal)	0.00 (0.00, 2.18)	0.00 (0.00, 3.22)	0.00 (0.00, 2.45)	0.00 (0.00, 1.98)	0.534
Nuts (g/1,000 kcal)	5.99 (0.00, 16.53)	0.00 (0.00, 16.07)	6.50 (0.00, 19.02)	7.91 (0.00, 15.81)	0.011
Yogurt (g/1,000 kcal)	0.00 (0.00, 77.07)	0.00 (0.00, 93.89)	21.20 (0.00, 80.56)	0.00 (0.00, 51.31)	0.003
Fish and seafood (g/1,000 kcal)	27.98 (0.00, 64.49)	35.39 (0.00, 78.96)	33.98 (0.00, 65.94)	17.38 (0.00, 46.87)	<0.001
Red meat (g/1,000 kcal)	44.76 (31.02, 62.59)	49.27 (32.87, 70.76)	45.98 (31.67, 62.79)	39.90 (29.12, 54.96)	<0.001
Processed meat (g/1,000 kcal)	0.00 (0.00, 0.00)	0.00 (0.00, 0.00)	0.00 (0.00, 0.00)	0.00 (0.00, 13.36)	<0.001
Saturated animal fat (g/1,000 kcal)^*^	0.00 (0.00, 0.00)	0.00 (0.00, 0.00)	0.00 (0.00, 0.00)	0.00 (0.00, 0.00)	0.029
Refined grains (g/1,000 kcal)	112.81 (89.78, 135.22)	105.54 (84.89, 125.31)	108.84 (89.10, 134.67)	125.74 (100.96, 141.79)	<0.001
Sugary beverages (g/1,000 kcal)	0.00 (0.00, 0.00)	0.00 (0.00, 0.00)	0.00 (0.00, 0.00)	0.00 (0.00, 0.00)	0.420
Ultra-processed food (g/1,000 kcal)	22.85 (0.00, 51.48)	20.35 (0.00, 51.31)	25.27 (0.00, 55.27)	24.99 (9.53, 47.61)	0.002

Values are presented as median (25th–75th percentiles). All dietary intakes were energy-adjusted using the density method (per 1,000 kcal). ^*^Indicates that the variable was zero in most samples, with a few non-zero extreme values unevenly distributed across groups, which explains the significant Kruskal-Wallis test result. DOS, dietary obesity-prevention score.

Further characterization of the inadequate energy intake subgroup was performed by examining the association between sociodemographic factors and DOS scores. Pregnant women with higher per capita monthly income had higher DOS scores, and the difference was statistically significant (*p* = 0.017). No significant differences in DOS scores were observed across categories of age, education, occupation, or family history of diabetes or hypertension (all *p* > 0.05).

### 3.2 Association of DOS and DASH scores with the risk of GDM in the total population and stratified by energy intake

In the total population, no significant association was observed between DOS and GDM risk (OR = 0.944, 95% CI: 0.879–1.015, *p* = 0.118). The interaction between DOS and energy intake status was not statistically significant (*p* for interaction = 0.193). Exploratory subgroup analyses were performed to further investigate the association between DOS and GDM risk within each energy intake stratum. As shown in Table 3 and Supplementary Figure S2, higher DOS was significantly associated with reduced GDM risk in the inadequate energy intake group (OR = 0.867, 95% CI: 0.773–0.973). No significant associations were observed in the adequate (OR = 1.042, 95% CI: 0.901–1.205) or excessive (OR = 0.974, 95% CI: 0.857–1.107) energy intake groups. An exploratory analysis was additionally performed to examine the association between the DASH score and the risk of GDM (Supplementary Table S2). In the inadequate energy intake group, DASH scores were not associated with the risk of GDM (DASH-7: OR = 1.011, 95% CI: 0.925–1.106, *p* = 0.804; DASH-8: OR = 1.000, 95% CI: 0.915–1.092, *p* = 0.995).

**Table 3:** Association between DOS and GDM risk stratified by energy intake status in the second trimester.

Energy status	*n*	Model 1		Model 2		Model 3	
		OR (95% CI)	*p*-value	OR (95% CI)	*p*-value	OR (95% CI)	*p*-value
Inadequate	729	0.889 (0.796–0.993)	0.037	0.869 (0.776–0.972)	0.014	0.867 (0.773–0.973)	0.015
Adequate	540	1.022 (0.890–1.174)	0.755	1.031 (0.896–1.188)	0.667	1.042 (0.901–1.205)	0.578
Excessive	625	0.978 (0.866–1.103)	0.714	0.976 (0.865–1.101)	0.692	0.974 (0.857–1.107)	0.687

Model 1 was adjusted for age, pre-pregnancy BMI. Model 2 was adjusted for age, education, per capita monthly income, pre-pregnancy BMI, occupation of pregnant woman, occupation of husband/partner. Models 3 was adjusted for age, education, per capita monthly income, pre-pregnancy BMI, energy intake during the second trimester, occupation of pregnant woman, occupation of husband/partner, family history of diabetes, and regular exercise before pregnancy.

### 3.3 Likelihood ratio tests examined the contribution of individual food groups to the DOS-GDM association

Likelihood ratio tests were performed to assess the contribution of each food group to the model fit. As shown in Table 4, excluding fruit from the model significantly worsened model fit (Δ-2LL = 16.091, df = 1, *p* < 0.001). In contrast, excluding any other food group (vegetables, plant to animal protein ratio, red meat, refined grains, or ultra-processed foods) did not significantly change the model fit (all *p* > 0.05).

**Table 4:** Likelihood ratio tests for the contribution of individual food groups to model fit.

Model	-2 Log Likelihood	Δ-2LL	df	*p*-value
Full model (all 6 food groups)	560.643	–	–	–
Model excluding fruit	576.734	16.091	1	<0.001
Model excluding vegetable	561.034	0.391	1	0.532
Model excluding plant-to-animal protein ratio	560.704	0.061	1	0.805
Model excluding red meat	561.125	0.482	1	0.488
Model excluding refined grains	560.685	0.042	1	0.838
Model excluding ultra-processed foods	561.164	0.521	1	0.471

### 3.4 Component analysis of DOS in relation to GDM risk in the inadequate energy intake subgroup

To identify the food groups contributing most to the observed association, a component analysis was performed using a mutually adjusted model. As shown in Table 5, fruit intake showed an inverse association with GDM risk (OR per 50 g/1,000 kcal = 0.905, 95% CI: 0.817–1.001, *p* = 0.053), whereas none of the other food groups reached statistical significance (all *p* > 0.05). To further examine whether the effect of fruit intake depends on overall diet quality, the inadequate energy intake subgroup was stratified by tertiles of DOS. As shown in Table 6, fruit intake was significantly associated with lower GDM risk in the middle DOS tertile (OR = 0.783, 95% CI: 0.620–0.990, *p* = 0.041) and showed a borderline significant association in the high DOS tertile (OR = 0.828, 95% CI: 0.686–1.000, *p* = 0.049). No significant association was observed in the low DOS tertile (OR = 1.071, 95% CI: 0.904–1.269, *p* = 0.430).

**Table 5:** Component analysis of DOS in relation to GDM risk in the inadequate energy intake subgroup (*n* = 729).

Food group	OR (95% CI)	*p*-value
Fruit (50 g/1,000 kcal)	0.905 (0.817–1.001)	0.053
Vegetables (g/1,000 kcal)	1.001 (0.999–1.003)	0.436
Plant-to-animal protein ratio	0.895 (0.572–1.400)	0.627
Red meat (g/1,000 kcal)	1.001 (0.994–1.009)	0.692
Refined grains (g/1,000 kcal)	1.003 (0.994–1.011)	0.560
Ultra-processed foods (g/1,000 kcal)	0.999 (0.992–1.005)	0.702

Model adjusted for age, pre-pregnancy BMI, energy intake during second trimester, education, per capita monthly income, occupation of pregnant woman and her husband, family history of diabetes, and regular exercise before pregnancy.

**Table 6:** Association between fruit intake and GDM risk stratified by tertiles of DOS in the inadequate energy intake subgroup (*n* = 729).

DOS level	*n*	GDM cases (%)	OR (95% CI)	*p*-value
Low DOS	212	47 (22.2)	1.071 (0.904–1.269)	0.430
Middle DOS	315	35 (11.1)	0.783 (0.620–0.990)	0.041
High DOS	202	31 (15.3)	0.828 (0.686–1.000)	0.049

Model adjusted for age, pre-pregnancy BMI, energy intake during second trimester, education, per capita monthly income, occupation of pregnant woman and her husband, family history of diabetes, and regular exercise before pregnancy. ORs represent the change in GDM risk per 50 g/1,000 kcal increment in fruit intake.

### 3.5 Dose-response relationship of fruit intake on GDM risk

**Figure 2:**
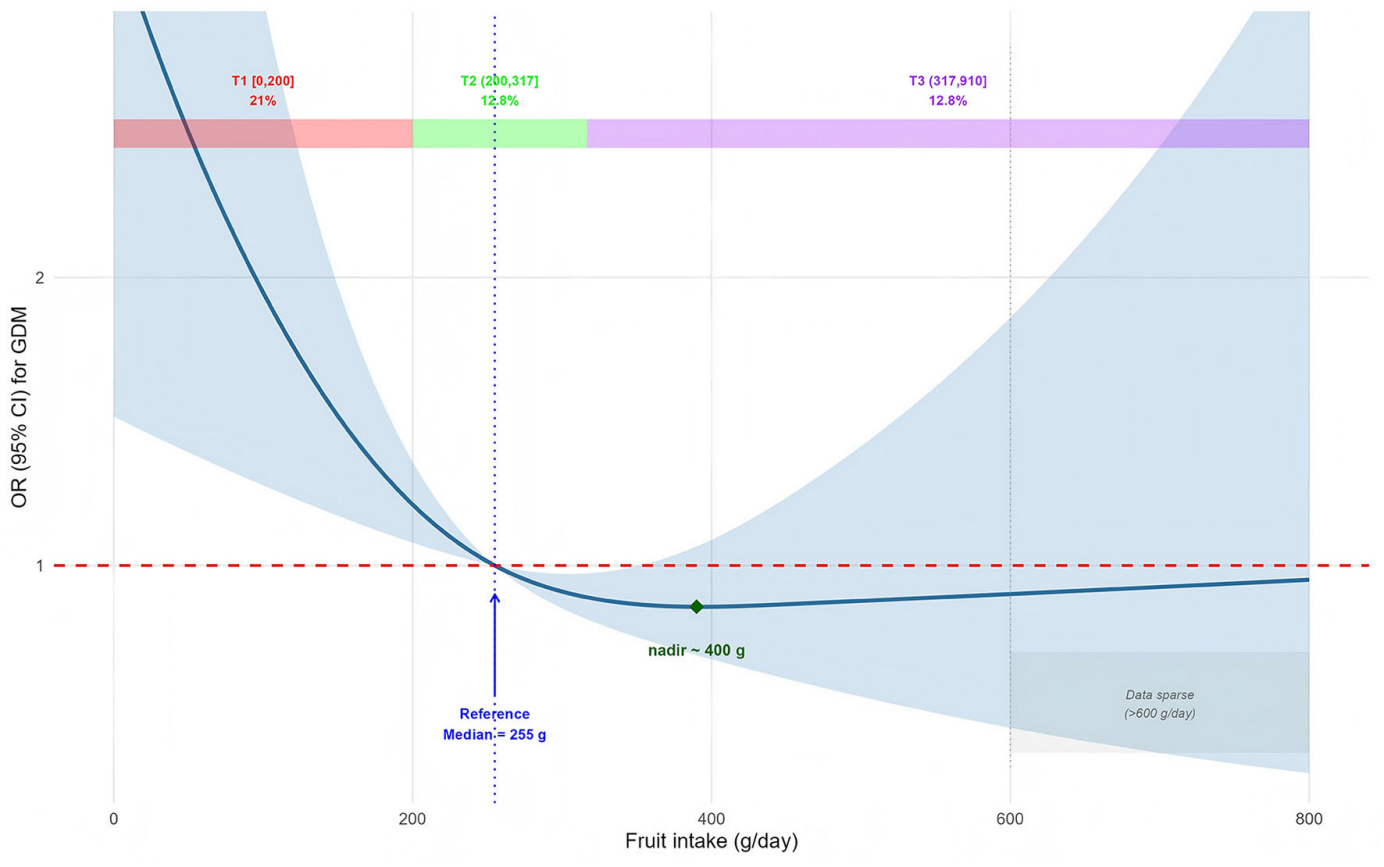
Dose-response relationship between fruit intake and GDM risk among pregnant women with inadequate energy intake. Odds ratio (solid line) and 95% CI (shaded area) estimated by restricted cubic spline regression (3 knots), with median intake (255 g/day) as reference. The green diamond indicates the approximate nadir (~400 g/day). Colored bars represent tertile distributions. Gray area indicates sparse data (>600 g/day). The model was adjusted for age, education, per capita monthly income, pre-pregnancy BMI, energy intake during the second trimester, occupation of pregnant woman and her husband, family history of diabetes, and regular exercise before pregnancy.

An exploratory dose-response analysis was conducted to examine the relationship between fruit intake and GDM risk in the energy-inadequate group. Restricted cubic spline analysis with 3 knots revealed a non-linear association between fruit intake and GDM risk (*p* for non-linearity = 0.048). Compared with the median intake (~255 g/day), the dose-response curve exhibited a non-linear pattern. Risk gradually decreased to a nadir at approximately 400 g/day, followed by a plateau with minimal rebound (Figure 2). The 95% CI widened substantially beyond 600 g/day, reflecting uncertainty due to sparse data. The inflection points were identified visually from the fitted spline curves and should be interpreted as descriptive features rather than precise thresholds. This trend was supported by the observed tertile data: participants in T1 [0, 200 g/day] had a GDM rate of 21.0%, whereas those in T2 (200, 317 g/day] and T3 (317, 910 g/day] both showed identical rates of 12.8%, suggesting that risk decreased substantially above 200 g/day and then stabilized, with no additional protective or adverse effect observed at higher intakes.

### 3.6 Sensitivity analysis for unmeasured confounding (E-value)

To assess the robustness of the observed association between fruit intake and GDM to unmeasured confounding, E-values were calculated. The values were derived from the effect size (OR = 0.896, 95% CI: 0.811–0.990) as estimated by multivariable logistic regression. The point estimate E-value was 1.48, and the E-value based on the upper limit of the 95% CI was 1.11. This implies that an unmeasured confounder would need to be associated with both fruit intake and GDM by a risk ratio of 1.48-fold (or 1.11-fold for the confidence limit) to fully explain away the observed association. These values suggest that the observed association is not highly robust.

## 4 Discussion

In this prospective cohort study, the association between the DOS and GDM risk was examined in Chinese pregnant women. No significant association was observed in the total population. The interaction between DOS and energy intake status was not statistically significant. However, *post hoc* subgroup analyses stratified by energy intake status showed that higher DOS was associated with reduced GDM risk among women with inadequate energy intake. These findings were hypothesis-generating and should not be viewed as definitive evidence for clinical recommendations. Likelihood ratio tests suggested that fruit intake may contribute to the stability of the DOS-GDM association. Dose-response analysis identified a non-linear relationship between fruit intake and GDM risk in the inadequate energy subgroup, with risk decreasing to approximately 400 g/day. After this point, the risk stabilized. This finding requires validation in independent cohorts.

In the energy-inadequate subgroup, women with lower DOS scores were more likely to have lower per capita monthly income, suggesting that sociodemographic factors may influence dietary choices during pregnancy. A review of 47 studies found that positive correlates of adherence to dietary guidelines during pregnancy, including age, education, employment, social class and certain medical histories (26). Another cross-sectional study showed that higher-income women had much better diet quality scores during pregnancy than middle- and low-income women (27). Together, these findings provide strong evidence that socioeconomic status has a general impact on dietary quality during pregnancy. These observations highlight the need for targeted nutritional interventions for less educated and economically disadvantaged women.

No significant association was found between DOS and GDM risk in the total population. Similar findings have also been reported in previous dietary pattern studies (28). This overall null finding may reflect divergent subgroup effects, whereby the protective association observed among women with inadequate energy intake was offset by the null effects in the adequate and excessive energy intake groups (29). The impact of dietary quality only becomes more apparent when energy intake is constrained. Additionally, the modest effect of dietary quality may be overshadowed by stronger determinants of GDM risk. These determinants include pre-pregnancy BMI, age, or residual confounding from unmeasured factors, which may dominate the risk profile in the overall population (30). Furthermore, individual genetic heterogeneity may also mask associations between dietary exposures and chronic disease risk (31, 32).

The lack of significant interaction between DOS and energy intake status may be partly attributable to the similar distribution of DOS scores across energy intake strata. This reflects the energy-adjusted nature of the score and the relatively homogeneous dietary quality in this cohort. Reduced variability resulting from the exclusion of rarely consumed foods may bias observed associations toward the null, particularly in interaction tests which require greater statistical power. One study reported that a highly skewed distribution of dietary patterns limited the ability to detect associations with GDM (33). In addition, interaction tests generally require larger sample sizes than main effect analyses. The modest sample sizes within each stratum may have limited the power to detect moderate or small interaction effects. Strong determinants of GDM risk, such as pre-pregnancy BMI, age, and education, may have overshadowed any modest effect modification by energy intake (34).

The finding that higher DOS was associated with reduced GDM risk only in the inadequate energy intake group may be explained by the modifying role of baseline energy status. When energy intake is sufficient or excessive, the metabolic impact of dietary composition may be overshadowed by the effects of total energy load. Evidence from the BORN2020 study indicated that energy intake exceeding the upper levels set by the European Food Safety Authority (EFSA), as well as higher intakes of protein, monounsaturated fatty acids, and dietary fiber during mid-gestation, although beneficial in balanced amounts, were associated with increased GDM risk when consumed beyond requirements (35). A randomized controlled trial further demonstrated that under energy-restricted conditions (1,200 kcal/day), dietary intervention significantly improved GDM outcomes and reduced the need for insulin therapy (36). Furthermore, in accordance with traditional Chinese culture, the majority of women follow a set of dietary precautions during the childbearing period. Consequently, many pregnant women avoid red meat and other high-energy foods, a pattern that may naturally lower total energy intake through food substitution (37). These observations suggest that when energy intake is constrained, the metabolic impact of diet quality becomes more discernible.

In the inadequate energy intake subgroup, DOS was significantly associated with GDM risk. The DASH score showed no such association (Supplementary Table S2). This discrepancy may be partly explained by the low consumption of certain DASH components in this cohort, including low-fat dairy, nuts, and legumes. Such an explanation is speculative. This should not be interpreted as evidence that DOS outperforms DASH. External validation in larger, independent cohorts is required.

Likelihood ratio tests suggested a possible contribution of fruit intake to the DOS-GDM association. Fruit intake was protective only in the middle and high DOS tertiles, indicating that its benefit depends on an overall healthy dietary pattern. This finding aligns with previous reports showing inverse associations between fruit consumption and GDM risk (38, 39). The beneficial effects of fruit may be related to nutritional components such as dietary fiber, vitamins, and phytochemicals (40, 41). A meta-analysis confirmed that anthocyanin supplementation significantly improved glycemic parameters and lipid profiles in individuals with type 2 diabetes, an effect attributed to their antioxidant and anti-inflammatory properties (42). In contrast, the plant to animal protein ratio was not associated with GDM risk, consistent with a prior study (10). The lack of independent effects for other food groups may suggest alternative pathways, such as anti-inflammatory or antioxidant mechanisms.

Dose-response analysis identified a non-linear relationship between fruit intake and GDM risk in the inadequate energy subgroup, with risk decreasing to approximately 400 g/day and stabilizing thereafter. The observed plateau may reflect statistical saturation rather than a true biological threshold, particularly given the widening confidence intervals beyond 600 g/day and the modest sample size. The apparent threshold should be interpreted as a descriptive feature of the fitted spline curve, not a precise clinical target.

To assess the robustness of the observed association between fruit intake and GDM to unmeasured confounding, E-values were calculated. The E-value analysis suggests that the observed inverse association between fruit intake and GDM is not robust to modest confounding. The confidence interval E-value of 1.11 is close to the null, implying that the lower bound of the effect estimate is sensitive to even weak confounding. This underscores the importance of considering potential residual confounding and highlights the need for future studies with more detailed covariate assessment. These hypothesis-generating findings may provide preliminary information for future research on fruit intake.

### 4.1 Strengths and limitations

This study had the following strengths. Firstly, the study included data from nine centers in China, covering diverse dietary patterns and significantly improving sample representativeness. Secondly, the use of the residual method and the energy density method for energy adjustment effectively improved the accuracy of dietary exposure assessment. Thirdly, multiple statistical methods were used to validate the results and sensitivity analysis were conducted, enhancing the credibility of the results. Finally, the prospective research design provided strong evidence for in-depth analysis of longitudinal associations in the real world.

However, this study also has several limitations. Firstly, although quality control measures were implemented, dietary data relies on self-report, which may result in recall bias and reporting bias. Secondly, the DOS was developed in non-pregnant adults. Its applicability during pregnancy requires further validation, though our findings offer preliminary support for its relevance. Thirdly, the final DOS was derived from this specific cohort, as rarely consumed food groups were excluded based on both distributional considerations and clinical feasibility. Therefore, external validation in independent cohorts is required. Fourthly, although adjustments have been made for key confounding factors, residual confounding from unmeasured factors such as genetic susceptibility may still affect the observed associations. Fifthly, the statistical power for detecting interactions was insufficient. Sixthly, the dataset did not permit reliable stratification by fruit type, processing method, or glycemic index. Fruit juice consumption was negligible in this cohort, and most fruits were low-GI whole fruits, precluding meaningful subgroup analysis by these characteristics. Finally, as all study participants were from China, the applicability of the results of this study to other populations may need further validation. In the future, large-scale and diversified cohort studies with comprehensive food composition data, extended follow-up time, and more objective dietary assessment methods are needed to verify the relationship between maternal diet and gestational diabetes, and clarify its potential mechanism.

## 5 Conclusion

In *post hoc* exploratory analyses, higher dietary quality may be associated with reduced GDM risk among pregnant women with inadequate energy intake, and fruit intake may play a role in this association. However, these findings require validation in independent cohorts. Future mechanistic research could include mediation analysis to explore the mediating role of specific nutrients or inflammatory pathways, or integrate genetic data to investigate gene-diet interactions. In addition, more objective dietary assessment methods, such as dietary biomarkers, could improve the accuracy of future studies.

## Data Availability

The data supporting the findings of this study are available from the Chinese Center for Disease Control and Prevention. Under the terms of our data use agreement, access is restricted to authorized members of the research team. Therefore, the data are not publicly available, and any third-party access would require separate permission from the Chinese Center for Disease Control and Prevention. Requests to access the datasets should be directed to: zengningli@hebmu.edu.cn.

## Supplementary material

Supplementary Figure 1 Validation of final DOS: correlation with total energy intake (*n* = 1,894).

Supplementary Figure 2 Forest plot of the association between DOS and GDM stratified by energy intake status.

Supplementary Table S1 Comparison of different DOS versions in association with GDM risk in the energy-inadequate group (n = 729).

Supplementary Table S2 Comparison of DOS and DASH in association with GDM risk.
