# Supplementary material for: Association between the dietary obesity-prevention score and risk of gestational diabetes mellitus: an exploratory subgroup analysis
Source: Front Nutr. 2026 Jul 24;13:1869787. doi: 10.3389/fnut.2026.1869787 (PMC13449703; doi:10.3389/fnut.2026.1869787)
Supplement: Supplementary file 1 [file Table_1.docx]

Supplementary Material

# 1 Supplementary Tables

**Supplementary Table S1.** Comparison of different DOS versions in association with GDM risk in the energy-inadequate group (n = 729).

| DOS version | Method | Components | Correlation with energy | OR (95% CI) | *p*-value |
| --- | --- | --- | --- | --- | --- |
| DOS1 | **Residual method** | Residual-based scores of all 13 food groups (vegetables, fruits, legumes, nuts, yogurt, fish and seafood, vegetable to animal protein ratio, red meat, processed meat, saturated animal fat, refined grains, sugary beverages, ultra-processed foods) | 0.295 | 0.970 (0.893-1.053) | 0.466 |
| **DOS2** | **Energy-density method** | All 13 food groups (same as above) | **-0.064** | **0.944 (0.875-1.018)** | **0.133** |
| DOS3 | **Energy-density method** | Excluding food groups with >50% of values not obtained: legumes,  processed meat, saturated animal fat, sugary beverages | -0.059 | 0.945 (0.875-1.021) | 0.153 |
| DOS4 | **Energy-density method** | Excluding food groups with >30% of values not obtained: nuts, yogurt, fish and seafood, plus those excluded in DOS3 | -0.131 | 0.897 (0.809, 0.993) | 0.037 |
| final DOS | **Energy-density method** | Change "vegetable to animal protein ratio" to "plant to animal protein ratio" | -0.088 | 0.867 (0.773-0.973) | 0.015 |

Note: All DOS versions were constructed based on tertile categorization of food group intakes (energy-density method) or residuals (residual method). The energy-density method calculates intake per 1000 kcal to adjust for total energy. Final DOS was selected as the primary score for subsequent analyses due to its robustness and parsimonious composition. Adjusted for age, education, Per capita monthly income, pre-pregnancy BMI, energy intake during the second trimester, Occupation of pregnant woman, Occupation of husband/partner, family history of diabetes, and Regular exercise before pregnancy. All models in the energy-inadequate group.

**Supplementary Table S2.** Comparison of DOS and DASH in association with GDM risk.

| Energy status | Score | Model 1 | | Model 2 | | Model 3 | |
| --- | --- | --- | --- | --- | --- | --- | --- |
|  |  | OR (95% CI) | *p*-value | OR (95% CI) | *p*-value | OR (95% CI) | *p*-value |
| Inadequate (n=729) | DOS | 0.889 (0.796-0.993) | 0.037 | 0.869 (0.776-0.972) | 0.014 | 0.867 (0.773-0.973) | 0.015 |
|  | DASH-7 | 1.054 (0.968-1.148) | 0.227 | 1.019 (0.933-1.113) | 0.677 | 1.011 (0.925-1.106) | 0.804 |
|  | DASH-8 | 1.045 (0.962-1.135) | 0.299 | 1.007 (0.924-1.098) | 0.873 | 1.000 (0.915-1.092) | 0.995 |
| Adequate (n=540) | DOS | 1.022 (0.890-1.174) | 0.755 | 1.031 (0.896-1.188) | 0.667 | 1.042 (0.901-1.205) | 0.578 |
|  | DASH-7 | 1.051 (0.946-1.166) | 0.352 | 1.061 (0.954-1.181) | 0.274 | 1.055 (0.943-1.180) | 0.349 |
|  | DASH-8 | 1.060 (0.961-1.169) | 0.244 | 1.072 (0.969-1.186) | 0.176 | 1.070 (0.963-1.189) | 0.207 |
| Excessive (n=625) | DOS | 0.978 (0.866-1.103) | 0.714 | 0.976 (0.865-1.101) | 0.692 | 0.974 (0.857-1.107) | 0.687 |
|  | DASH-7 | 1.006 (0.912-1.109) | 0.896 | 1.007 (0.912-1.109) | 0.896 | 1.009 (0.909-1.120) | 0.86 |
|  | DASH-8 | 0.977 (0.891-1.070) | 0.619 | 0.979 (0.892-1.073) | 0.651 | 0.978 (0.886-1.079) | 0.659 |

Note: The DASH score was constructed using 8 components: fruits, vegetables, nuts/legumes, whole grains, low-fat dairy (yogurt), sodium, red/processed meat, and sugary beverages. Energy-density method was applied for energy adjustment (intake per 1000 kcal). Tertile scoring (1–3) was used for all components, with positive scoring for beneficial components (fruits, vegetables, nuts/legumes, whole grains, yogurt) and reverse scoring (3–1) for adverse components (red/processed meat, sugary beverages, sodium). Two versions were calculated: DASH-7 (excluding sodium) and DASH-8 (including sodium).
